# Brain-machine interfacing control of whole-body humanoid motion

**DOI:** 10.3389/fnsys.2014.00138

**Published:** 2014-08-05

**Authors:** Karim Bouyarmane, Joris Vaillant, Norikazu Sugimoto, François Keith, Jun-ichiro Furukawa, Jun Morimoto

**Affiliations:** ^1^Computational Neuroscience Laboratories, Department of Brain Robot Interface, Advanced Telecommunications Research Institute International (ATR)Kyoto, Japan; ^2^Laboratoire d'Informatique de Robotique et de Micro-électronique de Montpellier, CNRS-University of Montpellier 2Montpellier, France; ^3^CNRS-AIST Joint Robotics Laboratory, UMI3218/CRT, National Intitute of Advanced Industrial Science and TechnologyTsukuba, Japan; ^4^National Institute of Information and Communications TechnologyOsaka, Japan; ^5^Graduate School of Frontier Biosciences, Osaka UniversityOsaka, Japan

**Keywords:** humanoid whole-body control, brain-machine interfacing, motor imagery, motion planning, semi-autonomous humanoid, contact support planning

## Abstract

We propose to tackle in this paper the problem of controlling whole-body humanoid robot behavior through non-invasive brain-machine interfacing (BMI), motivated by the perspective of mapping human motor control strategies to human-like mechanical avatar. Our solution is based on the adequate reduction of the controllable dimensionality of a high-DOF humanoid motion in line with the state-of-the-art possibilities of non-invasive BMI technologies, leaving the complement subspace part of the motion to be planned and executed by an autonomous humanoid whole-body motion planning and control framework. The results are shown in full physics-based simulation of a 36-degree-of-freedom humanoid motion controlled by a user through EEG-extracted brain signals generated with motor imagery task.

## 1. Introduction

Due to their design that allows them to be readily used in an environment that was initially arranged to accommodate the human morphology, that makes them more acceptable to the users, and easier to interact with, it is generally admitted that humanoid robots are an appropriate choice as living assistants for the everyday tasks, for instance for the elderly and/or reduced-mobility people. The problem that naturally arises is that of the control of such an assistant and how to communicate the wills and intentions of the user to the robot. This problem is of course general but becomes more challenging when addressing the above-mentioned category of users for which communication capabilities can also be impaired (stroke patients for example). This brings our initial idea of considering brain-machine interfaces (BMI) as the possible communication tool between the human and the humanoid assistant. Notwithstanding, brought along with this reflection was the more general question, non-necessarily application-directed, of a human using its brain motor functions to control a human-like artificial body the same way they control their own human body. This question becomes our main motivation and concern in the present work since solving it would pave the way of the discussed applicative perspectives. We thus propose our solution to it in this paper.

The approach we choose to investigate deals with the following constraints of the problem. First, we only consider easy-and-ready-to-use non-invasive BMI technologies. Among this class of technologies, we aim more specifically at the one that would align best and most intuitively with our expressed desire of mimicking human motor-control function, namely motor-imagery-based BMI, consisting ideally for the human user of imagining a movement of their own body for it to be replicated in the humanoid body, though we do not reach that ideal vision restricting our study for the sake of feasibility demonstration to the use of a generic motor-imagery task (imagining arm movement) that we re-target to the specific motion of the robot at hand (leg motion of the robot). Finally, the control paradigm for the humanoid robot we set as objective in our study is that of low-level joint/link-level control, to keep as general behavior and class of movements as possible for the user to replicate at the robot, without restriction of the class of movements allowed by particular higher-level humanoid motion controllers.

We address the related work and existing proposed solutions for this problem or approaching ones in the next section (Section 2). We then detail our own solution, based on the integration of, for the humanoid motion control part, an autonomous contact-based planning and control framework (Section 3), and for the BMI part, a motor-imagery-task-generated brain-signal classification method (Section 4). The integration of these two originally independent components is discussed in Section 5, 6 presents an example proof-of-concept experiment with a fully physics-simulated humanoid robot. Section 7 concludes the paper with discussion and future work.

## 2. Related work and proposed solution

**Figure 1:**
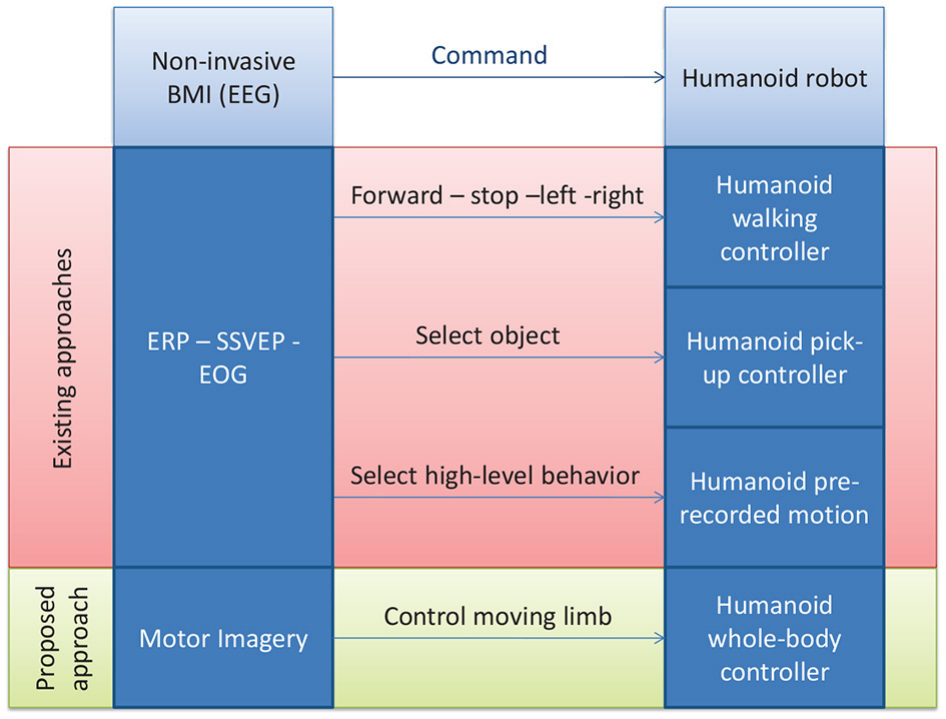
**A schematic illustration of the proposed approach vs. the existing ones for controlling a humanoid robot with non-invasive BMI**.

Various approaches have been proposed to solve the problem we stated in the introduction of controlling a humanoid robot with BMI (Bell et al., 2008; Bryan et al., 2011; Chung et al., 2011; Finke et al., 2011; Gergondet et al., 2011; Ma et al., 2013). All approaches, ours included, are based on the integration of a BMI technology with a humanoid controller, and can thus be categorized according to which strategy is followed for each of these two components. See Figure 1 for an overview.

From the BMI point-of-view, all these works do abide by our posed constraint of using non-invasive BMIs that rely on electroencephalography (EEG), generally utilizing the well-established frameworks of visual-stimulation-based event-related potentials (ERP) such as P300 in Bell et al. (2008), evoked potentials (EP) such as the steady state visually evoked potential (SSVEP) in Bryan et al. (2011); Chung et al. (2011); Gergondet et al. (2011), or hybrid approaches combining electrooculogram (EOG) with ERP such as in Ma et al. (2013), or P300 with motor-imagery-evoked event-related desynchronization (ERD) (Finke et al., 2011; Riechmann et al., 2011). None, however, investigated a solely motor-imagery-based BMI as stated in our motivations of replicating intuitive human motor-control strategies. Hence our first contribution in the integration initiative.

We adapt in this work a motor-imagery decoding scheme that we previously developed for the control of a one-degree-of-freedom robot and for sending standing-up/sitting-down commands to a wearable exoskeleton (Noda et al., 2012). It allows us to generate a three-valued discrete command that we propose to map to a one-dimensional subspace of the multi-dimensional whole-body configuration space motion of the humanoid, and more precisely the motion along a generalized notion of “vertical axis” of the moving end-limb, such as the foot of the swing leg in a biped motion for instance. As we detail in the course of the paper (Section 5), the motivation behind this strategy is to allow the user to assist the autonomous motion that might lead the moving limb to be “blocked” in potential field local minima while trying to avoid collision. The strategy can in future work be developed into a more sophisticated two-dimensional continuous command one as proven possible by recent and ongoing studies on motor-imagery control (Wolpaw and McFarland, 2004; Miller et al., 2010).

From the humanoid controller point-of-view now, the most standard retained solution consists in using available humanoid high level controllers. These can be either walking controllers with the commands “walk forward” “stop” “turn left” “turn right” sent to a walking humanoid, effectively reducing the problem of humanoid motion control to that of walk steering control (Bell et al., 2008; Chung et al., 2011; Finke et al., 2011; Gergondet et al., 2011), or an object selecton/pick-up controller, where the user selects an object in the scene and then the arm reaching/grasping controller of the robot picks up the desired object (Bryan et al., 2011). Finally Ma et al. (2013) use a hybrid control strategy where both walk steering and selecting a high-level behavior among a finite library can be done by switching between EOG and ERP control. With these strategies, a humanoid can be seen as an arm-equipped mobile robot, with wheels instead of legs (as it is actually the case in Bryan et al., 2011 where only the upper body is humanoid), and consequently the considerable amount of work done on BMI wheelchair control, for example, can be readily adapted. However, in doing so, the advantages of using a legged device over a wheeled one are partially lost, and we can no longer claim the need for the humanoid design nor defend the argument of the possibility of using the robot in everyday living environment which would present non-flat structures, such as stairs for example, with which the walking controllers are not efficient to deal.

While we admit that these strategies relying on walking pattern generators can in the long term benefit from the developments in these techniques that would allow them to autonomously cope with unstructured terrain (variable height stairs, rough terrain) (Takanishi and Kato, 1994; Hashimoto et al., 2006; Herdt et al., 2010; Morisawa et al., 2011), and that they can as well use the hierarchical architectures in which they are embedded as it is the case in Chung et al. (2011); Bryan et al. (2011); Ma et al. (2013) for switching, for example, to an appropriate stair-climbing controller when facing stairs, we choose in this work to investigate an entirely different approach that does not incorporate any kind of walking or high-level controller. Instead, we propose to allow the user to perform lower-level joint/link level control of the whole-body motion of humanoid, driven again by the desire of replicating the human low-level motor-control strategies into the humanoid, but also by the belief that a generic-motion generating approach will allow the robot assistant to deal more systematically with unpredictable situations that inevitably occur in everyday living scenarios and for which the discussed hierarchical architectures would not have exhaustively accounted. This is our second contribution. To achieve this goal, we rely on the contact planning paradigm that we previously proposed for fully autonomous robot (Bouyarmane and Kheddar, 2012), adapting it here to the instance of a BMI-controlled robot.

## 3. Humanoid controller

Our humanoid controller is based on the multi-contact planning paradigm, introduced in Hauser et al. (2008); Bouyarmane and Kheddar (2012). This controller allows for autonomously planning and executing the complex high-degree-of-freedom motion of the humanoid from a high-level objective expressed in terms of a desired contact state to reach. The controller works in two stages: an off-line planning stage and an on-line execution stage.

At the planning stage (Bouyarmane and Kheddar, 2011a), a search algorithm explores all the possible contact transitions that would allow the robot to go from the initial contact state to the desired goal contact state. What we mean by contact transition is either removing one contact from the current contact state (e.g., removing the right foot from a double-support state to transition to a left-foot single-support one) or adding one contact to the current contact state (e.g., bringing the swing right foot in contact with the floor to transition from a left-foot single-support phase to a double support phase). One must however note that a contact is defined as a pairing between any surface designated on the cover of the links of the robot and any surface on the environment, and is not restricted to be established between the soles of the feet and the floor surface. For instance, a contact can be defined between the forearm of the robot and the arm of an armchair, or between the palm of the hand of the robot and the top of a table. This strategy stems from the observation that all motions of humans can be broken down to such a succession of contact transitions, be it cyclic motions such as walking where these transitions occur between the feet and the ground, or more complex maneuvers such as standing up from an armchair were contacts transitions occur between various parts of the body (hands, forearms feet, tights, etc.) and various parts the environment objects (armchair, table floor, etc.). This feature makes our planning paradigm able to cope with situations that are broader than the ones classically tackled by humanoid motion planner that either plan for the motion assuming a given contact state (e.g., planning a reaching motion with the two feet fixed on the ground) (Kuffner et al., 2002; Yamane et al., 2004; Yoshida et al., 2006, 2008), or planning footprint placements assuming a cyclic walking pattern will occur on these footprints (Kuffner et al., 2001; Chestnutt et al., 2003, 2005). This aligns well with our initially expressed objective of controlling whole-body motion of any kind without restriction to a subclass of taxonomically identified motions.

At the above-described contact-transition search stage, every contact state that is being explored is validated by running an inverse-kinematics solver which finds an appropriate whole-body configuration (posture) of the robot that meets the desired contact state, while at the same time satisfying physics constraints to make the posture physically realizable within the mechanical limits of the robots (Bouyarmane and Kheddar, 2010). At the end of the offline-contact planning stage, we are provided with a sequence of feasible contact transitions and associated transition postures, that go from the initial contact state to the the goal.

The second stage of the controller is an on-line real-time low-level controller (Bouyarmane and Kheddar, 2011b) that will successively track each of the intermediate postures fed by the off-line planning stage, until the last element of the planned sequence is reached. The controller is formulated as a multi-objective quadratic program optimization scheme, the objectives being expressed in terms of the moving link of the robot involved in the current contact transition being tracked along the sequence (e.g., the foot if the contact transition is a sole/floor one), the center of mass (CoM) of the robot to keep balance, and the whole configuration of the robot to solve for the redundancies of the high-DOF motion. These objectives are autonomously decided by a finite-state machine (FSM) that encodes the current type of transition among the following two types:

- Removing-contact transition: the motion of the robot is performed on the current contact state, and the step is completed when the contact forces applied on the contact we want to remove vanish. This is done by shifting the weight of the robot away from the being-removed contact, tracking the CoM position of the following configuration in the sequence. There is no end-link motion in this kind of step. The corresponding FSM state is labeled “Shift CoM.”
- Adding-contact transition: the motion of the robot is performed on the current contact state, and the motion of the link we want to add as a contact is guided to its desired contact location. There is thus an end-link motion (contact link) in this kind of step. Balance is ensured by also tracking the CoM position of the following configuration in the sequence. The corresponding FSM state is labeled “Move contact link.”

As an example, a cyclic walking FSM state transition sequence will look like: Move contact link (left foot) → Shift CoM (on the left foot) → Move contact link (right foot) → Shift CoM (on the right foot) → Move contact link → … But non-cyclic behaviors are also possible and allowed, for example when standing up from an armchair where contacts between the hands of the robot and arms of chair can be added in succession and removed in succession.

The final output of the quadratic program optimization scheme is a torque command that is sent to the robot at every control iteration, after the execution of which the state of the robot is fed-back to the controller.

## 4. BMI decoding

Our aim is for the humanoid system to be controlled by using brain activities in the similar brain regions that are used to control the user's own body. Therefore, we asked a subject to control the simulated humanoid system by using motor imagery of arm movements so that brain activities in motor-related regions such as the primary motor cortex can be used.

As non-invasive brain signal acquisition device we use an electroencephalogram (EEG) system (64 channels and sampling rate of 2048 Hz). The brain signals are decoded and classified using the method that was applied and presented in our previous work (Noda et al., 2012), based on the spectral regularization matrix classifier described in Tomioka and Aihara (2007); Tomioka and Muller (2010). We recall the method here.

The EEG signals, of covariance matrices **C** considered as input, are classified into two classes, labeled with the variable *k*, with the following output probabilities (at sampled time *t*):

(1) $$P(k_{t}=+1|C_{t})=\frac{1}{1+\exp(-a_{t})},$$

(2) $$P(k_{t}=-1|C_{t})=\frac{\exp(-a_{t})}{1+\exp(-a_{t})},$$

with the logit being modeled as a linear function of **C**

(3) $$a_{t}=\text{tr}[W^{⊤}C_{t}]+b,$$

and where **W** is the parameter matrix to be learned (*b* is a constant-valued bias).

To learn **W** the following minimization problem is solved

(4) $$\min\sum_{t=1}^{n}\ln(1+\exp(-k_{t}a_{t}))+\lambda \|W{\|}_{1},$$

λ being the regularization variable (λ = 14 in the application below) and

(5) $$\left\|W{\|}_{1}=\sum_{i=1}^{r}\sigma_{i}[W\right]$$

being the spectral *l*_1_-norm of **W** (*r* is the rank of **W** and σ_*i*_[**W**] its *i*-th singular value).

Once the classifier learned, the 7–30 Hz band-pass-filtered measured EEG signals are decoded online, by down-sampling them from 2048 to 128 Hz, and applying Laplace filtering and common average substraction to remove voltage bias. Their covariance matrix, initialized at **C**_*t*_ = **x**^⊤^_*t*_**x**_*t*_ for the first time step *t* = 1, where **x**_*t*_ ∈ ℝ^1×64^ denotes the filtered EEG signals, are updated at every time step as follows

(6) $$C_{t}=\frac{1}{N}x_{t}^{⊤}x_{t}+\frac{N-1}{N}C_{t-1},$$

and used to compute the probabilities in Equations (1) and (2).

Finally, the three-valued discrete command *c*_*t*_ that is sent to the robot is selected from these probabilities through the following hysteresis

(7) $$c_{t}=\{\begin{array}{ll} +1 & \text{if }P(k_{t}=+1|C_{t})>P_{\text{thresh}}\text{ and }c_{t-1}\neq +1,\\ -1 & \text{if }P(k_{t}=-1|C_{t})>P_{\text{thresh}}\text{ and }c_{t-1}\neq -1,\\ 0 & \text{otherwise}, \end{array}$$

where the threshold is set at *P*_thresh_ = 0.6.

## 5. Component integration

**Figure 2:**
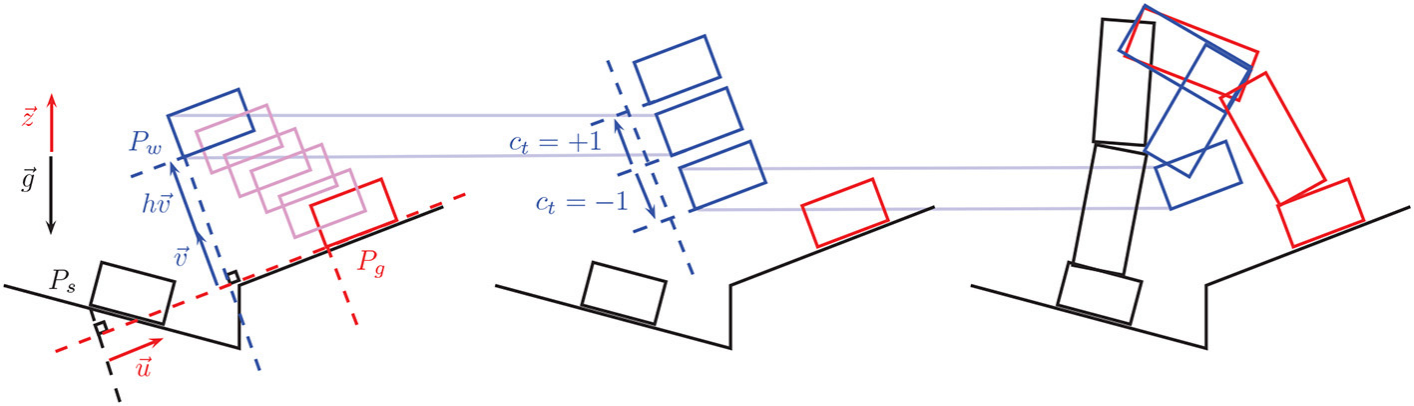
**The way-point moving strategy**. The rectangles in the left and middle figures represent positions of the moving foot (say the right foot, supposing the left foot is the support foot that is fixed and not represented here). In the right figure the whole leg motion is reconstructed from the foot motion. In all three figures, in black is the initial position of the foot/leg at the beginning of the step, in blue the controlled way-point position of the foot/leg at the middle of the step, and in red is the planned final foot/leg position at the end of the step. The left figure shows how a default position of the way point is initialized autonomously by a translation of the final planned position. $\vec{g}$ is the gravity vector, $\vec{z}$ the vertical unit vector (opposite to $\vec{g}$), $\vec{u}$ is the unit vector from the initial to the goal position along the goal planned-contact surface plane, $\vec{v}$ is the generalized vertical direction unit vector, i.e., the unit vector normal to $\vec{u}$ and in the plane defined by $\vec{u}$ and $\vec{z}$, finally, *h* is a pre-set default height. The middle figure shows how the way-point position is controlled via the command *c*_*t*_ sent through the motor imagery interface. Finally the left figure shows how the resulting motion of the leg actually looks like with the foot going through the desired way-point that was translated downwards via the command *c*_*t*_ = −1.

The command *c*_*t*_ devised in Equation (7) is sent to the online humanoid whole-body controller via UDP protocol at 128 Hz frequency and used to modify the planned and autonomously executed motion of the humanoid robot as described below and as schematically represented in Figure 2.

When the robot is executing a step that requires moving a link to a planned contact location (contact-adding step, executed by the state “Move contact link” of the FSM, see Section 3), then instead of tracking directly the goal contact location, we decompose the motion of the end-link (the contact link, for instance the foot) into two phases:

- Lift-off phase: The link first tracks an intermediate position located at a designated way-point.
- Touch-down phase: The link then tracks its goal location in the planned contact state sequence.

This two-phase decomposition allows the link to avoid unnecessary friction with the environment contact surface and to avoid colliding with environment features such as stairs.

Each of these two phases correspond to a sub-state of the meta-state “Move contact link” of the FSM, namely:

- State “Move contact link to way-point”
- State “Move contact link to goal”

Additionally, in order to avoid stopping the motion of the contact link at the way-point and to ensure a smooth motion throughout the step, we implemented a strategy that makes the transition from the former to the latter sub-state triggered when the contact link crosses a designated threshold plan along the way, before reaching the tracked way-point.

A default position of the intermediate way-point is automatically pre-set by the autonomous framework using the following heuristic (see Figure 2, left): Let *P*_*s*_ denote the start position of the contact link (at the beginning of the contact-adding step) and *P*_*g*_ denote its goal position (its location in the following contact state along the planned sequence). Let $\vec{g}$ denote the gravity vector, $\vec{z}$ the unit vector opposite to $\vec{g}$, i.e., $\vec{z}=-\vec{g}/\|\vec{g}\|$, and $\vec{u}$ the unit vector from *P*_*s,g*_ (*P*_*s*_ projected on the goal-contact surface plane) to *P*_*g*_, i.e., $\vec{u}=\vec{P_{s,g}P_{g}}/\|\vec{P_{s,g}P_{g}}\|$. Finally let $\vec{v}=\vec{u}\times (\vec{z}\times \vec{u})$ be the unit vector normal to $\vec{u}$ that lies in the plan defined by $\vec{u}$ and $\vec{z}$. The default way-point *P*_*w*_ is defined as

(8) $$P_{w}=P_{g}-\frac{1}{2}\vec{P_{s,g}P_{g}}+h\vec{v},$$

where *h* is the hand-tuned user-defined parameter that specifies the height of the steps. The command *c*_*t*_ in Equation (7) that comes from the BMI decoding system is finally used to modify in real-time this way-point position *P*_*w*_ by modifying its height *h* (see Figure 2, middle). Let δ*h* denote a desired height control resolution, then the modified position of the way-point through the brain command *c*_*t*_ becomes

(9) $$P_{w}(c_{t})=\{\begin{array}{ll} P_{g}-\frac{1}{2}\vec{P_{s,g}P_{g}}+(h+c_{t}\delta h)\vec{v} & \text{if }t=1,\\ P_{w}(c_{t-1})+c_{t}\delta h\vec{v} & \text{if }t>1. \end{array}$$

The command *c*_*t*_ could have been used in other ways, however we identified two principles that should in our view stand in a BMI low-level control endeavor of humanoid motion such as ours:

- Principle 1: The full detailed motion, that cannot be designed joint-wise by the BMI user, should be autonomously planned and executed from high-level (task-level) command.
- Principle 2: The brain command can then be used to locally correct or bias the autonomously planned and executed motion, and help overcome shortcomings inherent to full autonomy.

The way-point is a key feature to be controlled according to these two principles as it helps surmount the main limitation of the autonomous collision-avoidance constraint expressed in the on-line quadratic-program-formulated controller described in Section 3. This collision-avoidance constraint, that had to be formulated as a linear constraint in the joint acceleration vector of the robot in order to fit within the quadratic-program formulation [adapting to this end the velocity-damper formulation (Faverjon and Tournassoud, 1987)], acts as a repulsive field, with the tracked way-point acting as an attractive field, on the contact link. The resultant field (from the superposition of these two fields) can display local extrema corresponding to equilibrium situations in which the link stops moving though without having completed its tracking task (see Figure 9). Manual user intervention, here through the brain command, is then necessary to un-block the motion of the link by adequately moving the tracked way-point. The brain command is thus used here for low-level correction of a naturally limitation-affected full-autonomy strategy.

## 6. Proof-of-concept experiment

**Figure 3:**
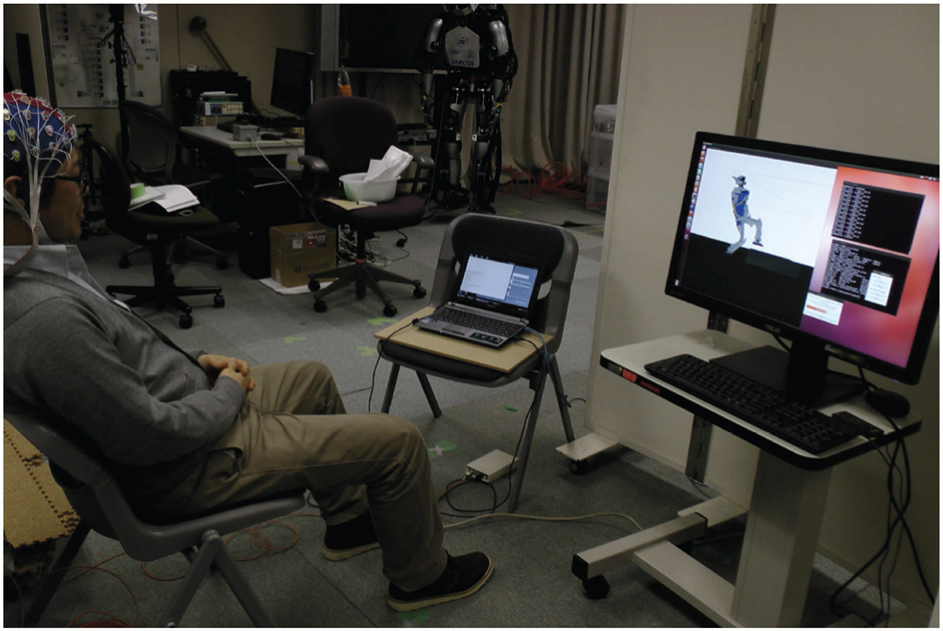
**Experiment setup**. The user is wearing an EEG cap. The laptop on his left side is used for decoding the motor imagery task signal, the computer on his right runs the real-time physics simulation allowing him to control the position of the moving foot through the visual feedback he gets from the simulator window.

The experiment we designed (see Figure 3 and video that can be downloaded at http://www.cns.atr.jp/~xmorimo/videos/frontiers.wmv) to test the whole framework is described as follows.

**Figure 4:**
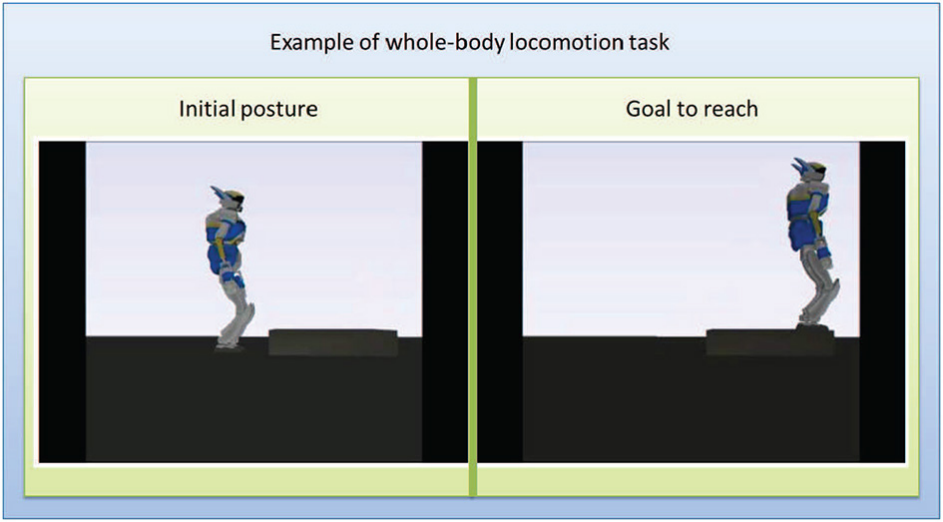
**Intial and goal positions for the experiment. Left**: initial configuration with the robot standing in front of the stair. **Right**: goal configuration with the robot standing at the extremity up on the srair.

An initial and goal configurations (Figure 4) are pre-specified manually by the user among a finite number of locations in the environment. In this case the initial configuration is standing in front of a stair and the goal task is to go up on the stair. This selection is for now done manually, but it can later also be selected through a brain command by embedding the strategy described in this work within a hierarchical framework such as the ones suggested in Chung et al. (2011); Bryan et al. (2011), that will switch between the behavior of selecting the high-level goal task and the low-level motion control.

**Figure 5:**
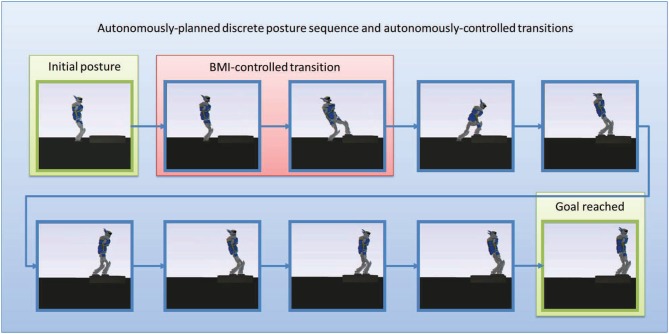
**The sequence of static postures planned autonomously**. The first posture is the initial posture. The second posture which looks like the first one keeps both feet on the ground but puts all the weight of the robot on the right foot so as to zero the contact forces on the right left to release it for the next posture. The third posture moves the now free left foot and puts it on the stair, but still without any contact force applied on it (all the weith of the robot is still supported by the right foot). the fourth postures keeps both feet at their current locations but shifts all the weight of the robot away from the right foot to put it entirely on the left foot, the right foot becomes free of contact forces, and so on.

Off-line, the framework autonomously plans the sequence of contact transitions and associated intermediate static postures to reach that goal (Figure 5), then the on-line controller is executed.

The user is wearing an EEG cap and is trained with 3 training sessions of approximately 5 min each to learn the parameter of the classifier described in Section 4, through a motor imagery task consisting of imagining respectively left arm and right arm circling movements for going up and down. This task is generic and we retained it since it gave us in our experiment better decoding performances than some other tasks (e.g., leg movements). The user has visual feed-back from the simulator on the desktop computer screen (on his right in Figure 3) and from a bar-diagram representing in real-time the decoded probability of the motor-imagery task classification on the laptop computer screen (on his left in Figure 3). The experiment was successfully completed on the first effective trial, which was the overall third trial (the first two trials were canceled after their respective training sessions since we encountered and fixed some minor implementation bugs before starting the control phase). The subject had prior experience with the same motor-imagery classifier in our previously-cited study (Noda et al., 2012). We only experimented with that one subject as we considered that we reached our aim of testing our framework and providing its proof-of-concept experiment.

The decoding of the BMI command is done in real-time and implemented in Matlab, and the brain command is then sent via UDP protocol to the physics simulator process implemented in C++.

**Figure 6:**
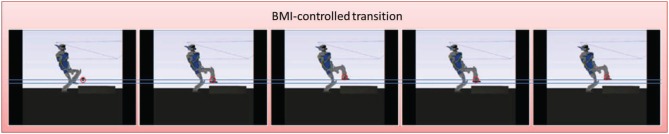
**The controlled motion**. The figures represent successive snapshots from the real-time controlled motion in the physics simulator. The controlled position of the way point appears in the simulator as a black sphere that we circle here in red for clarity. This position is tracked by the foot (more precisely at the ankle joint) throught the simulation. The two horizontal lines represent the level of the sole of the foot at the two positions sent as a command by the user through the BMI. These lines do not appear in the simulator we add them here only as common visualization reference lines for all the snapshots. In the first two frames the robot tracks the default position of the way point. In the third frame the user decides to move that position up, then down in fourth frame, and finally up again in the fifth frame.

We tested the way-point control strategy in the second step of the motion (the first contact-adding step along the sequence, the highlighted transition in Figure 5). Figure 6 focuses on this controlled part of the motion. The user controlled the position of a black sphere that represents the position of the targeted way-point, that the foot of the robot tracks in real-time, while autonomously keeping balance and avoiding self-collisions, joint limits, and collision with the environment. A total of 8 commands (“up”/“down”) were sent during this controlled transition phase, that we voluntarily made last around 300 s (5 min) in order to allow the user to send several commands. We then externally (manually) triggered the FSM transition to the following step along the sequence and left the autonomous controller complete the motion without brain control. That autonomous part was completed in about 16 s. See the accompanying video.

**Figure 7:**
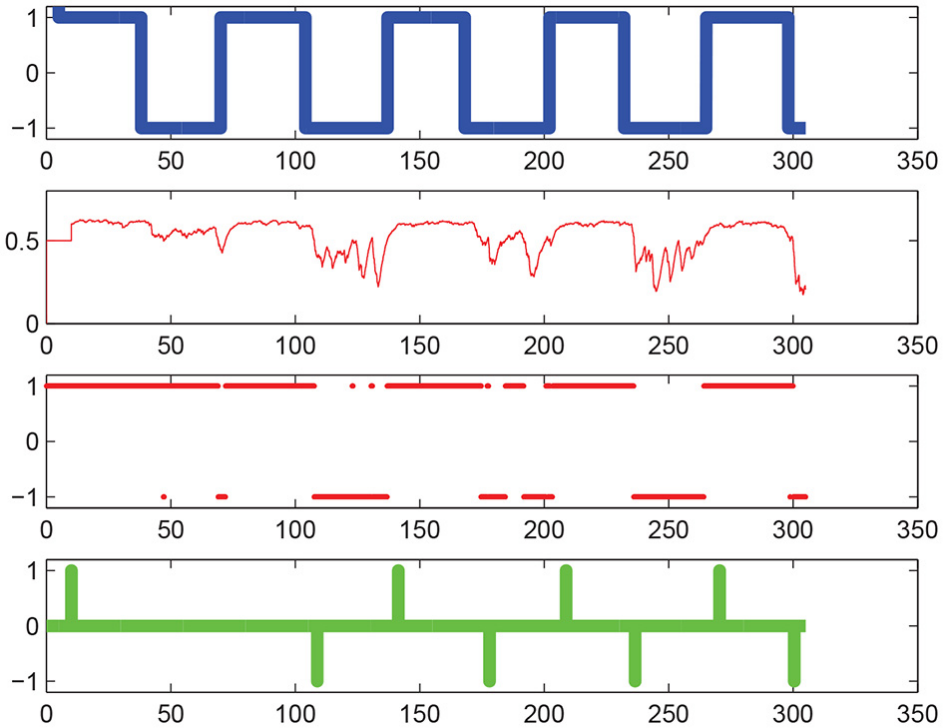
**Motor imagery decoding performances**. On the horizontal axis is iteration number. From top to bottom: the thick blue line represents the command cue given as an input to the user, the thin red line represents the decoded brain activities [the probability *P*(*k*_*t*_ = +1|**C**_*t*_)], the thick red point markers represent the estimated classified label [*P*(*k*_*t*_ = +1|**C**_*t*_) ≥ 0.5 or < 0.5], finally the thick green line represents the command *c*_*t*_ sent to the robot (based on the threshold *P*_thres_ = 0.6). Note that this green command does not represent the position of the way-point but the instantaneous rate of change in this position between two successive time steps *t* and *t* + 1, according to Equation (9), line 2 (i.e., the “derivative” were we talking of a continuous and differentiable function rather than the time-discretized one at hand).

**Figure 8:**
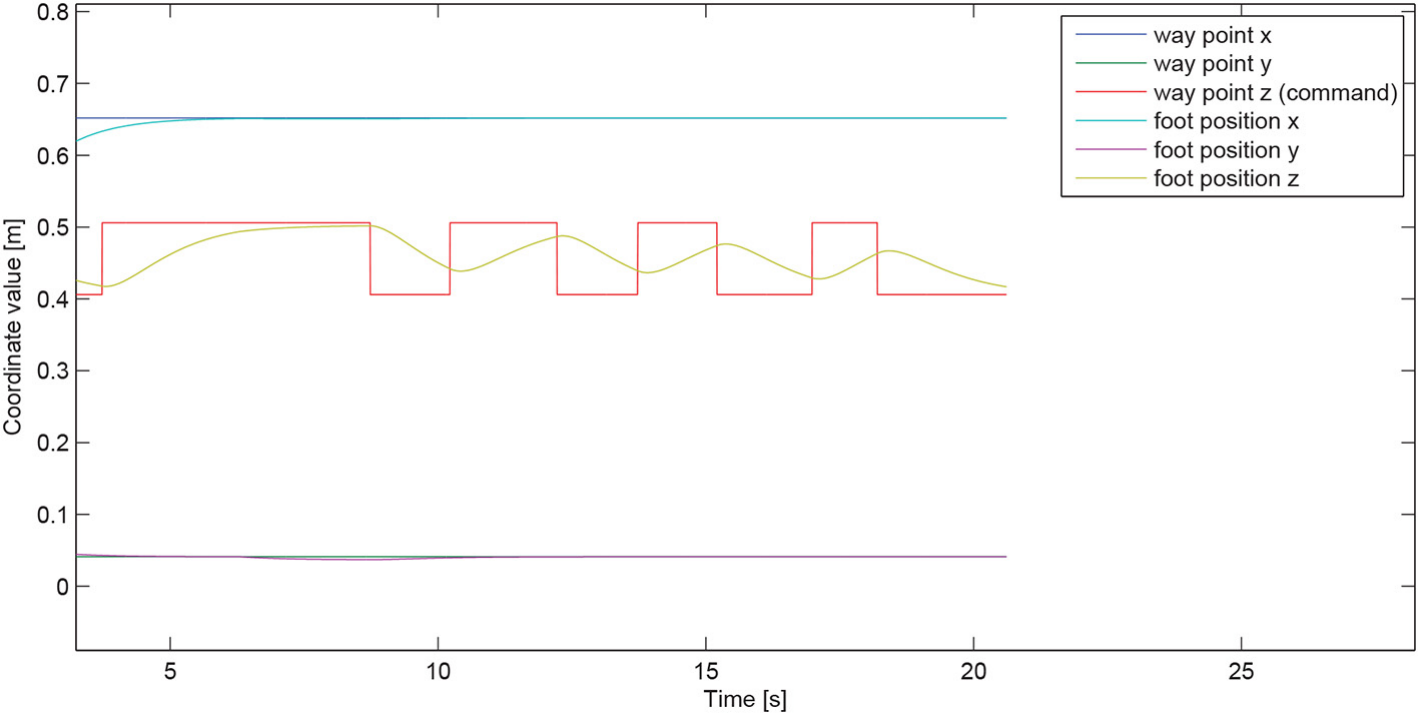
**Way-point tracking performance**. The user-controlled quantity, that happens to be in the particular case demonstrated here the *z*-coordinate of the tracked way-point (the “generalized” vertical direction being reduced in this case to the “conventional” vertical direction, meaning $\vec{v}$ ≡ $\vec{z}$ in Figure 2, since the goal-contact surface on the stair is horizontal), is represented by the piecewise-constant red curve. The corresponding motion of the foot, that tracks this command-induced way-point position, is shown in yellow curve. the two other coordinates of the foot (*x* and *y*) are auonomously maintained by the controller at the corresponding ones of the way-point and stay at their desired values throught the command phase.

Figure 7 illustrates the decoding performances of the BMI system, while Figure 8 shows the tracking performance of the humanoid whole-body controller. The table below gives computation time figures executed on a Dell Precision T7600 Workstation equipped with a Xeon processor E5-2687W (3.1 GHz, 20 M). Full details on the physics simulator, including contact modeling and resolution, and collision detection, can be found in Chardonnet et al. (2006); Chardonnet (2012).

**Table:** 

Offline planning	2.7 s
Average online control command (QP) (@ 200 Hz)	2.661 ms
Average online simulation step (@ 1 kHz)	0.389 ms
BCI classifier training and learning session	~ 30 min
Average online BCI signal buffering (@ 2048 Hz)	0.137 ms
Avg online BCI classification (@ 128 Hz) no control signal sent (*c_t_* = 0)	0.204 ms
Avg BCI classification (@ 128 Hz) control signal sent (*c_t_* = +1 or −1)	6.20 ms

**Figure 9:**
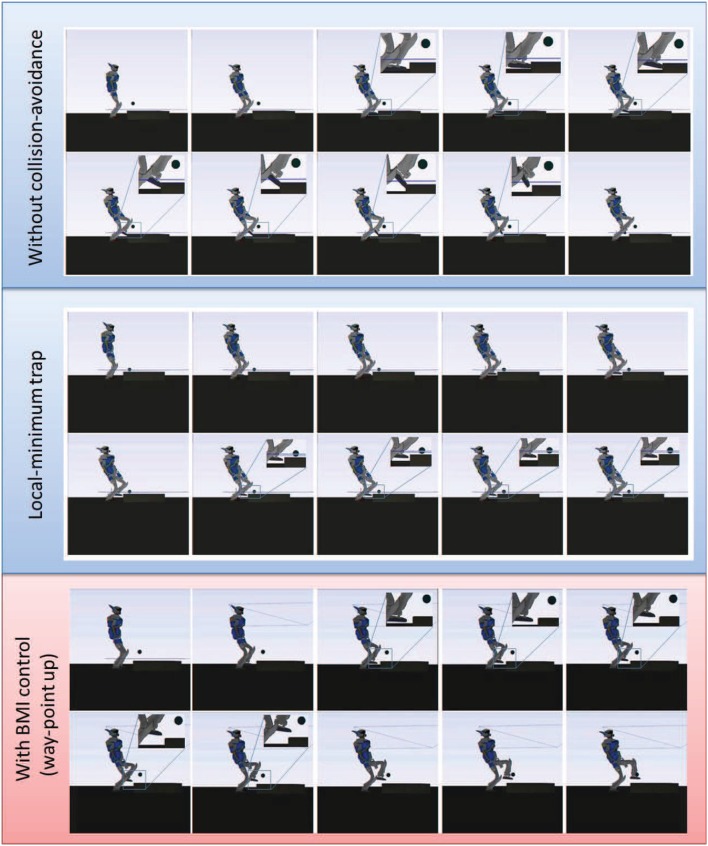
**Comparison of the controlled transition motion in three instances. Top**: without collision-avoidance constraint, the foot of the robot collides with the stair while targeting its goal, and the simulation stops. **Middle**: with autonomous collision-avoidance constraint that happens to create in this case a local-minimum trap, the robot reaches an equilibrium situation and stays idle for as long as we let the simulation run (infinite time). **Bottom**: The autonomous collision-avoidance strategy combined with the proposed BMI-control approach helps reposition the way-point and overcome the local-minimum problem. The robot safely reaches the goal contact location and the motion along the sequence can be completed.

From this experiment, we confirmed that the autonomous framework can be coupled with the BMI decoding system in real-time in simulation and that the simulated robot can safely realize the task while receiving and executing the brain command.

## 7. Discussion and future work

This work demonstrated the technical possibility of real-time online low-level control of whole-body humanoid motion using motor-imagery-based BMI.

We achieved it by coupling an existing EEG decoder and whole-body multi-contact acyclic planning and control framework. In particular, this coupling allowed us to control a one-dimensional feature of the high-DOF whole-body motion, designed as the generalized height of moving link way-point, in a discrete way. Though the motor-imagery task used in our proof-of-concept experiment was a generic one (left-arm vs. right-arm circling movement), we plan in the future to investigate more specific motor-imagery tasks that are in tighter correspondence with the limb of the robot being controlled, along the longer-term user's-mind-into-robot's-body “full embodiment” quest that motivates our study as expressed in our introductory section. Since previous studies reported that imagery of gait and actual gait execution have been found to recruit very similar cerebral networks (Miyai et al., 2001; La Fougère et al., 2010), we may be able to expect that a human can control a humanoid the same way they control their own human body through motor imagery.

We also aim now at continuous control of two-dimensional feature of this whole-body motion, allowing not only the control of the tracked way point but also of a corresponding threshold plan that decides when to trigger the transition between the lift-off and touch-down phases. We believe this can be achieved based on the previous work done for example on motor-imagery two-dimensional cursor control (Wolpaw and McFarland, 2004). Other previous studies also discussed the possibilities of using EEG for such continuous control (Yoshimura et al., 2012). In addition, for the continuous two-dimensional feature control, explicit consideration of individual differences in cerebral recruitment during motor imagery may be necessary (Meulen et al., 2014). As a future study, we may consider using transfer learning approaches (Samek et al., 2013) to cope with this individual difference problem.

Finally, we aim at porting this framework from the simulation environment to the real robot control, so that in future study we may possibly use the proposed framework in a rehabilitation training program to enhance recovery of motor-related nervous system of stroke patients.

### Conflict of interest statement

The authors declare that the research was conducted in the absence of any commercial or financial relationships that could be construed as a potential conflict of interest.
